# A Model for the Self-Organization of Vesicular Flux and Protein Distributions in the Golgi Apparatus

**DOI:** 10.1371/journal.pcbi.1003125

**Published:** 2013-07-18

**Authors:** Iaroslav Ispolatov, Anne Müsch

**Affiliations:** 1 Departamento de Física, Universidad de Santiago de Chile, Santiago, Chile; 2 Department of Developmental and Molecular Biology, Albert Einstein College of Medicine, New York, United States of America; University of York, United Kingdom

## Abstract

The generation of two non-identical membrane compartments via exchange of vesicles is considered to require two types of vesicles specified by distinct cytosolic coats that selectively recruit cargo, and two membrane-bound SNARE pairs that specify fusion and differ in their affinities for each type of vesicles. The mammalian Golgi complex is composed of 6–8 non-identical cisternae that undergo gradual maturation and replacement yet features only two SNARE pairs. We present a model that explains how distinct composition of Golgi cisternae can be generated with two and even a single SNARE pair and one vesicle coat. A decay of active SNARE concentration in aging cisternae provides the seed for a *cis* 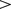 *trans* SNARE gradient that generates the predominantly retrograde vesicle flux which further enhances the gradient. This flux in turn yields the observed inhomogeneous steady-state distribution of Golgi enzymes, which compete with each other and with the SNAREs for incorporation into transport vesicles. We show analytically that the steady state SNARE concentration decays exponentially with the cisterna number. Numerical solutions of rate equations reproduce the experimentally observed SNARE gradients, overlapping enzyme peaks in *cis*, medial and *trans* and the reported change in vesicle nature across the Golgi: Vesicles originating from younger cisternae mostly contain Golgi enzymes and SNAREs enriched in these cisternae and extensively recycle through the Endoplasmic Reticulum (ER), while the other subpopulation of vesicles contains Golgi proteins prevalent in older cisternae and hardly reaches the ER.

## Author Summary

We have developed a quantitative model to address a fundamental question in cell biology: How does the Golgi apparatus, an organelle composed of multiple cisternae that exchange vesicles, steadily maintains its inhomogeneous protein composition in the face of ongoing cisternal aging and replacement, and cargo entry and exit. We do not assume any *a priori* polarity within the Golgi apparatus or directionality of vesicular traffic. The Golgi cisternae inevitably lose active proteins that specify vesicle fusion, the SNARE molecules, as they age, thus breaking the symmetry between compartments and establishing the “seed” for directional vesicular transport. This small decrease in SNARE concentration in older cisternae is then further self-enhanced by the progressively more directional vesicular transport of SNAREs. Competition of enzymes for incorporation into predominantly retrograde-fusing vesicles in turn generates overlapping but distinct stationary enzyme peaks. Applying these general mechanisms of fusion asymmetry and competitive vesicle loading to the actual situation in the stacked mammalian Golgi, we reproduced the experimentally observed distributions of the two SNARE pairs that operate in the Golgi, and enzyme peaks in *cis*, medial and *trans* cisternae. We believe that our study attempts the first self-consistent explanation for the polarity in the Golgi stack.

## Introduction

The Golgi apparatus is composed of multiple compartments, called cisternae, typically 6–8 in mammalian cells. The individual cisternae are enriched in glycosylation and other enzymes, which form distinct but overlapping gradients with peaks in the *cis*, medial or *trans* cisternae [1].

**Figure 1:**
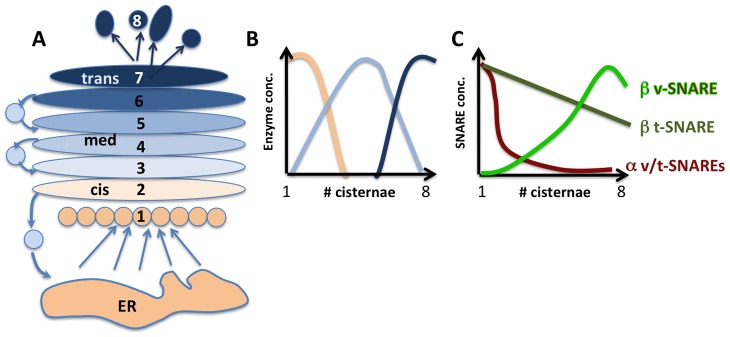
Schematic representation of a stacked Golgi apparatus that undergoes cisternal maturation. A) ER-derived vesicles (beige) fuse with each other to yield the first, most *cis*, cisterna. Individual cisterna mature from position 1 to position 8, where they disintegrate into transport carriers destined for the plasma membrane and endosomes. Vesicles originating from cisterna #2 deliver *cis* Golgi proteins to cisterna #1 while at the same time cisterna #2 receives Golgi resident proteins from cisterna #3. B) The cisternae are categorized as *cis*, medial and *trans* based on the abundance of Golgi residence proteins, mostly glycosylating enzymes, which exhibit distinct but overlapping peaks along the Golgi stack according to their sequential role in the processing of exocytic cargo. C) Two SNARE pairs, which we term 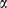 SNARE (purple) and 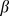 SNARE (green) are thought to mediate intra-Golgi transport of resident proteins. The respective v and t-SNAREs of 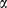 SNARE both decay with a steep gradient from *cis* to *trans*. 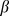-t-SNAREs decay with a shallow gradient, while its corresponding 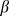-v-SNARE concentration increases from cisternae 1 to 8. The graphs are schematic representations of data from [17].

As anterograde cargo traverses the Golgi apparatus from *cis* to *trans*, it becomes modified by Golgi enzymes in an assembly-line fashion. Efficient and correct cargo processing depends on the distribution of glycosidases, glycosyltransferases and other enzymes within the different Golgi sub-compartments in their expected order of function [2]. Several mechanisms for cargo movement through the Golgi apparatus have been proposed. Of those, the cisternal maturation hypothesis is best supported by all available experimental data [3], [4]. According to this concept, cargo enters the Golgi by fusion of Endoplasmic Reticulum (ER)-derived vesicles with each other that form a new cisterna at the *cis* face of the Golgi. The cargo exits the Golgi in transport carriers that emerge from the *trans* most cisterna when it disintegrates, thus maintaining the Golgi apparatus at a steady state. Individual cisternae mature by shedding their characteristic Golgi enzymes and at the same time acquiring Golgi resident proteins from the more *trans* cisterna [5], [6] (Fig. 1A).

It has been shown that Golgi resident proteins shuttle between the cisternae in vesicles [7], [8], [9]. But how do individual cisternae acquire and maintain their specific and distinct enzyme compositions via vesicular transport while the Golgi apparatus undergoes maturation?

Glick et al. provided one piece of explanation with a simple model according to which competition of Golgi proteins for incorporation into retrograde-destined vesicles accounts for their sorting within the Golgi cisternae [10]. Proteins that are good competitors are efficiently removed from the maturing cisternae and accumulate in the *cis* Golgi while proteins that are poor competitors can only enter vesicles after the good competitors have been depleted, and thereby end up in more *trans* cisternae. While this model explains steady enzyme segregation, it is based on an unexplained premise, namely, that the Golgi-enzyme containing vesicles preferentially fuse with the younger rather than the older cisternae.

Fusion of vesicles with acceptor membranes is specified by Soluble N-ethyl-maleimide-sensitive factor Attachment protein Receptors (SNAREs), integral membrane proteins that reside in the vesicle and target membrane [11], [12]. They function according to a key-lock principle: Cognate SNAREs form a four-helical bundle, with one chain contributed by a R-SNARE on one membrane and one heavy and two light chains provided by corresponding Q-SNAREs on the opposite membrane to pull donor and acceptor membranes close enough to fuse [13]. Theoretical work by Heinrich and Rapoport has shown that sets of compatible SNAREs with preference for incorporation into a specific type of coated vesicle can spontaneously generate and maintain non-identical compartments [14] when each compartment features a specific pair of compatible SNAREs and corresponding vesicle type. The Golgi however, maintains its 6–8 compartments with only 2 cognate SNARE pairs and one type of vesicle (COPI) [15]. How is this accomplished? A higher concentration of SNARE complexes in younger compared to older cisternae could readily explain the preference for retrograde fusion of COPI vesicles, which in turn can yield the differential enzyme peaks as described by Glick et al. [10]. A *cis*-to- *trans* decrease is indeed observed for Golgi Q-SNAREs [15] (Fig. 1C). But how are these SNARE gradients established in the first place? To complicate matters, a R-SNARE implicated in intra-Golgi traffic forms a counter-current gradient with increasing levels from *cis*-to- *trans*, [16] [17], (Fig. 1C). How is this compatible with retrograde transport?

We present a model of inter-cisternal vesicular transport in which we do not assume any *a priori* asymmetry within the Golgi apparatus. The transport is mediated by 2 cognate SNARE pairs, which compete with each other and with other Golgi residents for incorporation into a single vesicle type. The retrograde directionality of vesicular flux is triggered by the temporal decrease of the concentration of cisternal SNAREs, which occurs via loss of SNARE-containing vesicles, including the recycling of COPI vesicles from the Golgi to the ER, decay, and inhibition of SNARE molecules. As a result, cisternal age becomes a distinguishing factor: *trans* cisternae are older than *cis* cisternae and thus contain fewer SNAREs. A small distinction in SNARE concentrations provides the seed for a *cis* 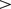 *trans* gradient, which becomes self-enhanced by vesicular transport of the SNAREs. The steady SNARE gradient controls a predominantly retrograde vesicular flux in which Golgi enzymes with stronger affinities for the coated vesicles cycle predominantly between the *cis* cisternae and the ER, while weaker-binding enzymes only enter vesicles from later cisternae and exhibit less ER retrieval.

## Results

### General features of the model

We assume that

1. The Golgi consists of a stack of 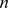 cisternae, which move in an anterograde direction or “mature”, carrying with them their SNAREs, enzymes (such as glycosyltransferases), and proteins that are being processed. The latter will not be considered here. Once every 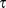 time units, a new cisterna is added to the *cis* end of the stack, while the most mature cisterna dissolves and disappears from the *trans* end of the stack. The new cisterna is formed by coalescence of ER-derived vesicles and contains fixed concentrations of SNAREs and enzymes.
2. Along with cisternal progression, vesicles containing SNAREs and Golgi enzymes continuously bud from each cisterna. We assume that the vesicles provide local transport and can only fuse with the neighboring *cis* (less mature) and *trans* (more mature) cisternae, and with the progenitor. Indeed, in the stacked mammalian Golgi, coil-coiled vesicular tethering factors which span the distance between adjacent cisterna are thought to grab vesicles even prior to their release from the donor cisterna and prevent them from reaching more distant cisternae [18], [19]. We will later relax this restriction and consider transport in a non-stacked Golgi, as it exists, for example, in the yeast *Saccharomyces cerevisiae*.
3. SNAREs and Golgi enzymes are uploaded into a vesicle via competitive binding to a fixed number of vesicular sites. We assume that the vesicular transport results primarily in the movement of cargo without any significant change in the volume and budding surface area of the cisternae. This is supported by observations that the size of all cisternae is similar [20] and our estimates that taking into account the vesicular transport of membrane itself would not significantly alter the results.

Functioning of the model hinges on two general principles: Establishment and maintenance of a directed retrograde vesicular flux and sorting of the vesicular cargo via competition for binding sites.

### Establishment of a *cis* 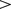 *trans* SNARE gradient that mediates retrograde vesicular flow

To reveal the universality of the proposed self-establishing mechanism of vesicular traffic directionality we first consider the simplest possible setup, a single cognate SNARE pair and vesicle type. We assume that the rate of vesicular fusion is proportional to the product of the concentrations of the SNAREs present in vesicles and cisternae, respectively. The precise nature of SNARE molecules does not have to be specified here. We can even consider the SNAREs as mere proxy for fusion-specifying factors. The probability for a vesicle to fuse with a given cisterna depends solely on the cisternal concentration of compatible SNAREs, and cisternae with higher SNARE concentration have a higher probability to absorb vesicles. A retrograde vesicular flux thus requires a *cis* 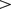 *trans* gradient in cisternal SNAREs.

We propose that key to a robust *cis* 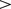 *trans* SNARE gradient is the observation that all systems, living and otherwise, function with a loss. As Golgi cisternae mature they inevitably lose active SNARE molecules. Such a decay of active SNAREs breaks the symmetry between the otherwise identical cisternae in a systematic way: The older *trans* cisternae contain less SNAREs than the younger *cis* cisternae. The SNARE loss can occur by escape of SNARE-carrying vesicles that fuse with the ER thus recycling their content. However, some of the cisternal SNARE decay is likely due to irreversible loss that requires some new SNARE synthesis to replenish the system.

**Figure 2:**
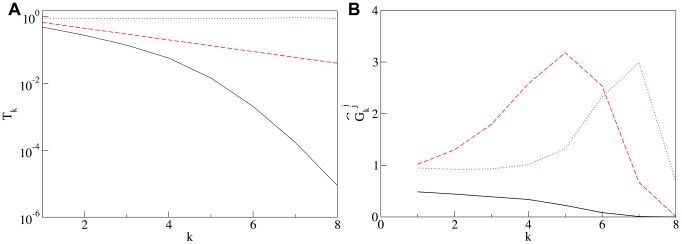
Self-generated concentrations of SNAREs and enzymes. **Panel A:** Steady state concentration of cisternal SNARE 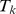 vs the number of cisterna 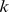 for: both the loss and the vesicular transport mechanisms are enacted (solid line), only the loss mechanism operates (dashed line), only vesicular transport occurs (dotted line). All concentrations are sampled immediately before the cisternal shift event, when the number of each cisterna is incremented by one. The definitions of parameters are given in Methods. Here and in all following plots it is assumed that 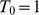 and 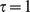, i.e. all concentrations are expressed in the units of initial concentrations and the time is expressed in units of the cisternal maturation period. Solid line: 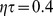 and 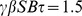, dashed line: 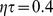 and 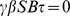, dotted line: 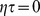 and 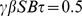, for all curves 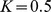. **Panel B:** Distribution of Golgi enzymes: *cis* (solid line), medial (dashed line) and *trans* (dotted line) established as a result of competition for incorporation into vesicles. Vesicular flux is controlled by the gradient of cisternal SNAREs shown by the solid line in the left panel, vesicles from the first cisterna can exit the Golgi and fuse with the ER. The parameters for the enzyme transport are 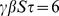, 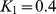, 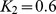, and 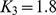.

The “seed” SNARE gradient generated in this manner sets a preference for vesicles to fuse with *cis* rather than *trans* cisternae, thus initiating the directed vesicular transport. As SNAREs are transported retrograde, their *cis* 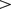 *trans* gradient is further enhanced. When the vesicular flux becomes balanced by the anterograde transport of SNAREs due to cisternal maturation, the system comes to a steady state. Indeed, we show both numerically and analytically that the seed gradient, created by the temporal decay of SNAREs, is self-enhancing (Figs. 2a and 5, which is presented in *Methods*, and Eqs. (11, 12, 13)). Importantly, while vesicular transport significantly increases the seed gradient produced by SNARE loss, without that loss vesicular transport by itself cannot produce or maintain any gradient, (see Eq. (13) and subsequent illustrations in Methods). This is in accordance with the results of [14] that the single SNARE pair/single coat minimal system cannot spontaneously break the initial symmetry of compartments. The constant progression of cisternae is equally important for maintaing the steady state SNARE gradient and directional vesicular flux. Without the progression, the seed SNARE gradient would have been equilibrated via vesicular transport.

We note that at steady state the vesicular flux does not depend on the concentration of SNAREs in the vesicles: Lower concentrations of vesicular SNAREs are compensated by a higher steady state number of vesicles. Naturally, a vesicle should contain a minimum number of vesicular SNARE molecules to ensure any fusion at all.

The calculation of the steady state SNARE gradient and vesicular flow are presented in the Methods section.

### Establishment of Golgi enzyme peaks in *cis*, medial and *trans* cisternae via SNARE-mediated retrograde vesicular traffic

**Figure 3:**
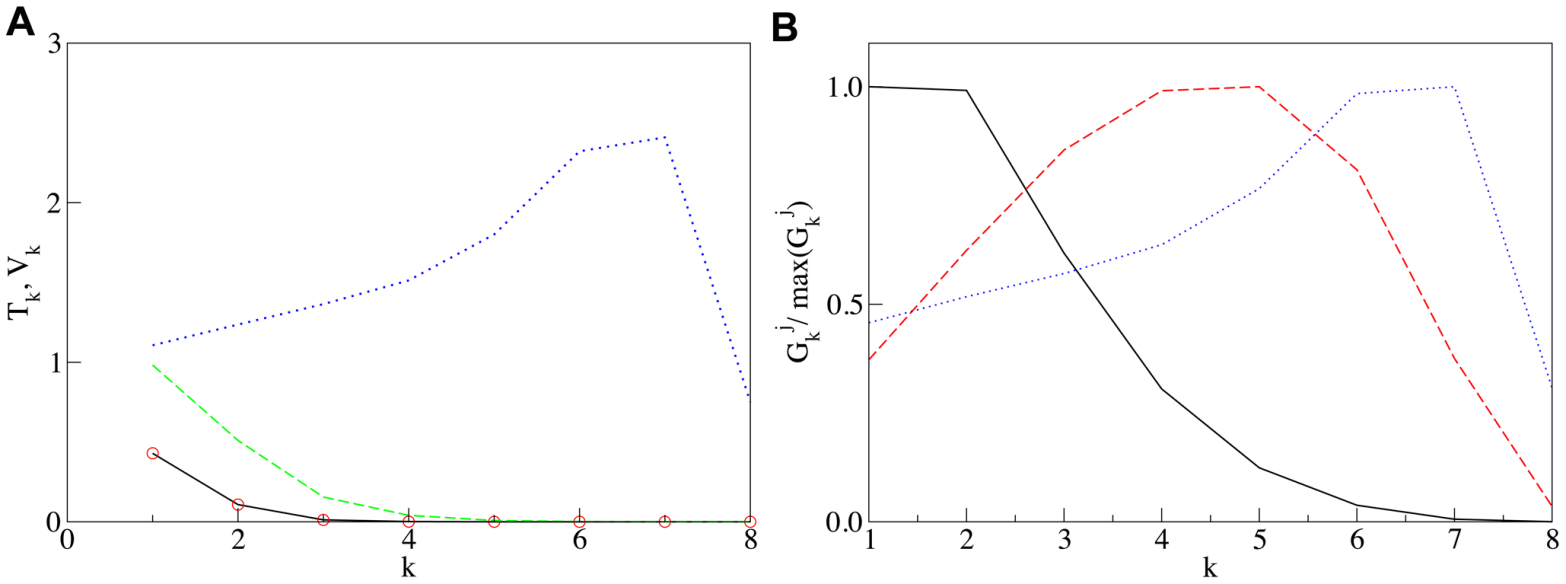
Self-generated steady state distributions of alpha and beta SNAREs and enzymes as it is observed in the Golgi apparatus. **Panel A:** 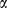 t-SNARE (solid line) and v-SNARE (circles) coinciding with t-SNARE, and 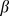 t-SNARE (dashed line) and v-SNARE (dotted line) vs. the cisternal number 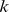. **Panel B:** cis (solid line), medial (dashed line) and trans (dotted line) Golgi enzymes normalized by their maximum value vs number of cisterna 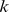. The parameters are: Decay rates 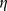 are zero for all substances except for 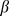 t-SNARE for which 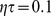, the vesicular transport coefficient 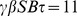, and dissociation constants for vesicular binding are 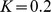 for ER v-SNARE, 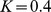 for cis t- and v-SNAREs, 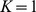 for trans t-SNARE, 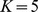 for trans v-SNARE, 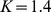 for cis enzymes, 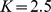 for median enzymes, and 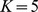 for trans enzymes. Initial concentrations of all substances in the first cisterna are 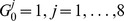, and the concentration of t-SNARE in ER is 0.7.

Next, we investigated how retrograde vesicular flow, created by the cisternal SNARE gradient, maintains the inhomogeneous steady state distribution of Golgi enzymes during cisternal maturation. To this end, we further developed the principle proposed by Glick et al. that attributes the different cisternal enzyme profiles to the competition of enzymes for the binding sites in vesicles [10]. For simplicity, we assume three categories of Golgi enzymes with peaks in *cis*, medial and *trans* cisternae, and with strong, intermediate and weak affinities for vesicular binding sites, respectively (Schematically depicted in Fig. 1B). Unlike in earlier models [10] and [21], the fraction of binding sites occupied by each type of enzyme is determined by mass action equilibrium. Also, in contrast to [10] and [21] where a number of *ad hoc* assumptions about vesicular flow were used, we “couple” the enzyme-carrying capacity to the self-established vesicular flow described above. Hence, while each vesicle competitively uploads enzymes according to their dissociation constants, its fusion probability is determined by the cisternal SNARE gradient shown by the black curve in Fig. 3A. To study the competition mechanism in its simplest form, we assume here that the SNARE distribution is unperturbed by enzyme uploading.

We find that the distribution of enzymes radically depends on whether vesicles originating from the first cisterna can exit the Golgi and fuse with its *cis* neighbor, the ER. If we permit ER recycling, the desired *cis*-medial- *trans* 3-mode steady state localization can be reproduced (Fig. 3B). Enzymes that have the highest affinity for the vesicular coat concentrate in the *cis* Golgi. A substantial fraction of these enzymes is loaded from the first cisterna into ER-bound vesicles and leaves the Golgi. In more central compartments, where *cis* enzymes are depleted, medial Golgi enzymes outcompete the weaker-binding *trans* enzymes for space in the vesicles. As a result, those enzymes advance with the maturing cisterna until the mid-Golgi where their concentration peaks, and then become effectively loaded into retrograde vesicles. Finally, the weak-binding enzymes can only incorporate into vesicles when all stronger-binding competitors are depleted. Their concentration peaks in the penultimate cisterna. The ultimate cisterna, equivalent to the disintegrating cisterna or *trans* Golgi network (TGN), exhibits a somewhat lower enzyme concentration as it does not receive any incoming retrograde vesicular traffic.

On the other hand, if none of the enzyme-carrying vesicles can escape to the ER, the cisternal distribution of all enzymes converges onto a single peak form (Fig. 6, presented in the *Methods* section). In this case the overall steady state abundance of enzymes increases with their affinity for vesicular binding: The stronger-binding enzymes are more efficiently retrieved to younger cisternae and thus better avoid being flushed out with the disintegrating *trans* cisterna than the weaker-binding enzymes. As a consequence of their higher concentration, the stronger-binding enzymes do not get sufficiently removed from younger cisternae to achieve their *cis*-Golgi peak and at the same time they do not give their weaker competitors any chance to enter the retrograde transport vesicles in the later cisternae. Hence, all enzymes peak at the *trans* face of the Golgi. Thus, recycling of enzymes to the ER is necessary for establishing the *cis*-medial- *trans* enzyme segregations. At the same time, we observe that the steady state SNARE distribution and resulting intra-Golgi vesicular flux is only weakly affected by the presence or absence of ER-recycling. This is because both scenarios feature an inherent loss mechanism, which breaks the intra Golgi conservation of SNAREs.

We also observe that, as discussed in [21], the competition-based enzyme segregation is rather sensitive to the variation of model parameters. Thus, it is possible that mechanisms have evolved to make the cisternal enzyme distribution more robust. One such mechanism, the change in enzyme affinity for vesicular binding sites with cisternal age, has been studied in [21] and could easily be incorporated into the a more detailed versions of our model.

The quantitative details of the calculation of the steady state enzyme concentrations, including Fig. 6, which illustrates enzyme distribution in the absence of ER-recycling, are presented in Methods.

### The two Golgi SNARE pairs can function with a single vesicle type to establish their own gradients and the observed Golgi enzyme peaks in *cis*, medial and *trans*

We now apply the general mechanisms of fusion asymmetry and competitive vesicle binding to explain the specific SNARE and enzyme distributions as they are actually observed in the mammalian Golgi. The important adjustment to our basic model is that the Golgi apparatus features not one, but two cognate SNARE pairs. The first pair, which we label 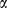, consists of the monomer SNARE rBet1 with its trimer SNARE partner Membrin/ERS24/Syntaxin5. The second pair, labeled 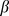, consists of the monomer SNARE GS15, compatible with the trimer SNARE complex of Gos28, Ykt6 and Syntaxin5. There is solid experimental evidence for both pairs to be incorporated in COPI vesicles [17], [22] and to participate in vesicular traffic of Golgi resident proteins [23], [24], [25].

To reproduce three Golgi enzyme peaks in concurrence with the experimentally observed distributions of the 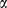 and 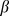 SNARE pairs we introduce an additional specification at this point, namely that the monomeric SNAREs rBet1 and GS15 mediate fusion only when present on the vesicle, and the trimeric-SNARE complexes only when present in the cisternae. In the following paragraph we provide a justification for the functional allocation of SNAREs as vesicular and cisternal.

In the Golgi, only the 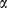 SNARE proteins actually have a *cis* 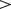 *trans* distribution [17] such as shown in Fig. 2A. The 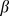 SNARE Gos28 also decreases from *cis* to *trans* [26]; however, its cognate monomeric SNARE partner GS15 accumulates in the *trans*-most cisternae instead [17], and the GS15 yeast homologue Sft1p is also enriched in the late Golgi [16], [23], (see Fig. 1C). If GS15 and the Gos28-Ykt6-Synt5 complex could both function as fusiogenic SNAREs in the cisternae, our model of vesicular flux would imply that Golgi enzymes known to depend on this SNARE pair for vesicular traffic undergo anterograde rather than retrograde transport. The anterograde vesicular enzyme transport does little to improve enzyme segregation as the cisternal maturation already moves enzymes in *trans* direction. More importantly, the anterograde vesicular transport makes the enzyme recycling impossible. Our allocation agree with *in vivo* observations: monomeric SNAREs act indeed most often as vesicle- or v-SNAREs and the trimeric SNAREs generally function at the target membrane (and are therefore typically referred to as t-SNAREs), [27]. But we also have a mechanistic explanation for why trimeric Golgi SNAREs function in the cisternae rather than the vesicles: When we consider the monomeric and trimeric SNAREs of a cognate SNARE pair separately, the SNARE that is most abundant in the vesicle determines which of the cisternal SNAREs the vesicle engages with. If the monomeric SNARE is more abundant in a vesicle than the trimeric SNARE, it will specify that the vesicle fuses with the cisterna which has the highest amount of cognate trimeric SNAREs, regardless of its monomeric SNARE concentration. Thus, when monomeric and trimeric SNARE partners differ significantly in their affinity for vesicles, the one with higher affinity becomes the v-SNARE, leaving the other to function in the cisternae. This is the case especially for the 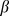 SNARE pair as Syntaxin 5, the limiting partner in both 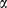 and 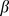 trimeric SNARE complexes, is at least 4 times less abundant than the 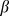 monomer GS15 in COPI vesicles (See Fig. 7B in [17]). Syntaxin-5's apparent poor affinity for Golgi vesicles explains its observed homogenous distribution in Golgi cisternae. However, the other constituents in 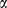 and 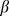 trimers are more efficiently transported by vesicles, thus maintaining the cisternal SNARE gradient. It follows from these observations that the two Syntaxin 5 containing trimeric Golgi SNAREs function as t-SNAREs.

In addition to the two Golgi SNAREs, we consider a third v-SNARE, which mediates the fusion of Golgi-derived vesicles with the ER. It is ERS24, which thus has a dual function as part of a t-SNARE complex in intra Golgi transport and as v-SNARE in Golgi-to-ER transport. The corresponding ER t-SNARE does not leave the ER and is therefore not considered here [15].

Apart from the SNARE specifications, we implemented a similar set of minimal assumptions as for the single SNARE scenario:

1. The rate of vesicular fusion with Golgi cisternae is determined by both 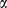 and 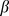 SNARE pairs and is proportional to the sum of the products of the cognate v-SNARE and t-SNARE concentrations.
2. All SNAREs and Golgi enzymes compete for the same binding sites in the vesicles. This is in agreement with the findings for ER-derived COPII vesicles, the only instance where cargo-competition for coat binding has been elucidated to date [28].
  However, our model reproduces the enzyme segregation as well in the case when the enzymes compete only with each other and not with SNAREs for vesicular binding sites, as shown in Fig. 2.
3. Vesicles fuse locally, i.e. with the *cis* and *trans* neighbors of the progenitor cisterna and the progenitor cisterna itself. In addition to fusing with Golgi cisternae, vesicles also fuse with the ER with a rate that is controlled by the product of the concentrations of the vesicular ER v-SNARE and a fixed concentration of ER t-SNAREs. Due to the expansive volume of the ER that brings it in proximity to the entire Golgi apparatus, we assume that all vesicles can fuse with the ER independent of their originating cisterna.
4. The age-dependent decrease (loss) of the cisternal concentration of the 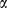 t-SNARE, which is essential for triggering the retrograde directionality of the traffic, occurs due to transport of vesicles to the ER. Therefore, no additional decay term is introduced for it. A small age-dependent decay term is introduced for the 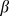 t-SNARE (see Eq. (19)).

We found a good qualitative agreement between our results and the experimentally observed concentration profiles. With the proper choice of dissociation constants (Table 1), 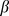 t-SNARE decay rate, and vesicular transport intensity, the model functions in the following way: The strong coat-binding affinities of 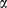 and ER SNAREs effectively package them into vesicles that bud from the younger *cis* cisternae. These vesicles have a high probability to fuse with the ER due to a substantial concentration of the ER SNAREs. These vesicles also recycle a good fraction of strong-binding *cis* enzymes, and a part of medial enzymes to the ER. The recycling of 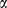 t-SNAREs to the ER seeds a cisternal gradient, which is responsible for the mostly retrograde direction of vesicular transport in the early cisternae. The recycling of 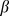 t-SNAREs to the ER is poor, yet when coupled with age-dependent decay, the 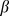 t-SNAREs extends the *cis* 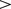 *trans* t-SNARE gradient to the *trans* Golgi. In more mature cisternae where the 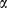 and ER SNAREs and *cis*-enzymes are depleted, the vesicles incorporate the weaker-binding molecules, such as medial and, to a lesser extent, *trans* enzymes and 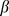 SNAREs. These vesicles have a much lower probability to reach the ER and transport their cargo mostly to younger Golgi cisternae. Finally, the *trans*-most cisternae bud vesicles that contain predominantly *trans* enzymes and 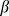 v-SNAREs. These cargoes are transported mostly retrograde, but hardly reach the ER. The total fraction of each protein that is retained in the Golgi (as compared to that recycled to the ER) can be appreciated by its concentration in the *trans*-most cisterna in Fig. 3B. Since the figures represent the situation before the last cisternal maturation step and removal of the last cisternae, the protein concentration that remains in the Golgi is equal to the initial concentration (set equal to one for all molecules), minus the loss to the ER.

**Table 1:** Dissociation constants for binding to vesicular sites that yield the plots depicted in Fig. 3.

Substance	Dissociation constant *K*
ER v-SNARE	0.2
*α* t-SNARE	0.4
*α* v-SNARE	0.4
*β* t-SNARE	1
*β* v-SNARE	5
*Cis* enzyme	1.4
Medial enzyme	2.5
*Trans* enzyme	5

Our prediction that *cis*-enzymes, 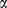 and ER SNAREs recycle through the ER at a higher level than 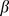 SNAREs and *trans* enzymes is indeed born out by numerous experimental observations in yeast and mammalian cells. *Cis* but not *trans* Golgi markers accumulated in the ER upon an acute ER-exit block [23], [29] or in the ER-derived intermediate compartment (ERGIC) after a temperature-induced exit block from this compartment [30], [24].

Based on the SNARE dissociation constants that yielded the experimentally observed protein gradients (Table 1) we further predict that monomeric ERS24, which functions as ER-v-SNARE, has the highest affinity of all SNAREs for the COPI coat, followed by the 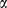 v- and t-SNAREs, (rBet1p and the proteins Syntaxin5 and Membrin, which together with ERS24 make up the 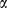 t-SNARE complex). Indeed, ERS24 is much higher concentrated in COPI vesicles than any of the other v-SNAREs (Fig. 8B in [17]). Syntaxin 5 is translated as a long and short version in mammalian cells [31]. The longer form features a known ER-retrieval signal and we predict that it is this form that predominantly functions in the 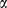 t-SNARE complex and is more efficiently incorporated into COPI vesicles then the short form that likely functions mostly as 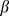 t-SNARE, which has a higher dissociation constant than the 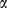 t-SNARE.

**Figure 4:**
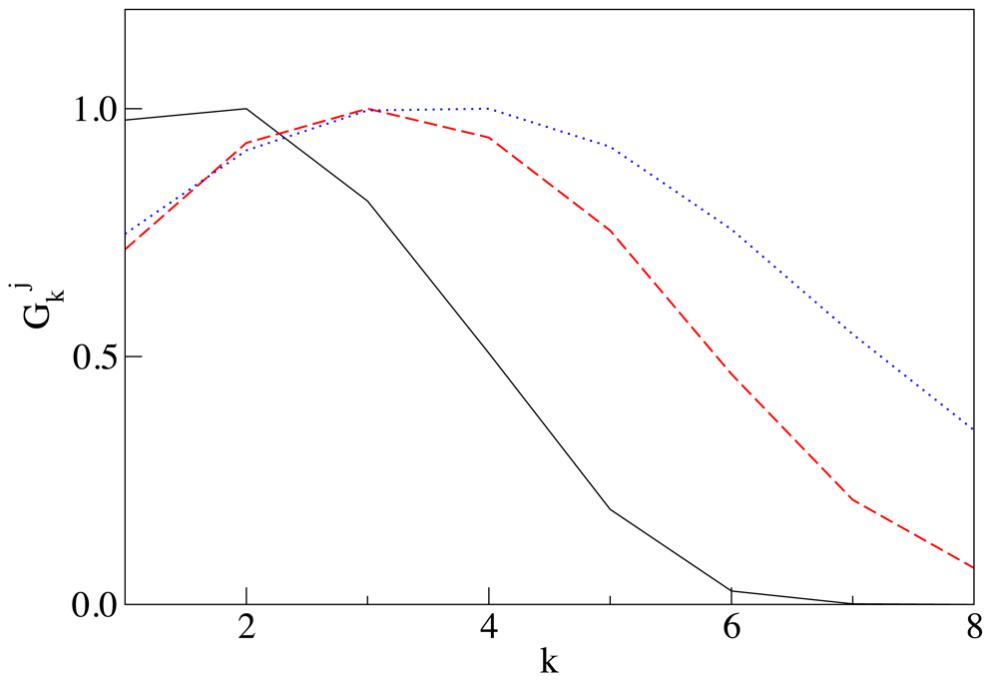
Steady state concentrations of three classes of enzymes in the unrestricted fusion scenario. Golgi enzyme concentrations are normalized by their maximum value. The parameters are: Decay rates for the *cis* and *trans* t-SNARE are 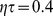, the vesicular transport coefficient 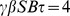, and dissociation constants for vesicular binding are: 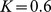 for the ER v-SNARE, 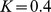 for the *cis* t- and v-SNAREs, 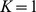 for *trans* t-SNARE, 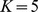 for *trans* v-SNARE, 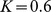 for *cis* enzymes, 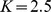 for medial enzymes, and 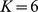 for *trans* enzymes. Initial concentrations of all proteins in the first cisterna are 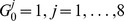, and the concentration of the t-SNARE in the ER is 0.7.

So far we assumed that vesicles only fuse with the immediate neighbors of their progenitor cisternae. A stacked Golgi, however, is not a requirement for Golgi asymmetry and cisternal maturation, which are also observed in *S. cerevisiae* where individual Golgi cisternae are scattered throughout the cytoplasm [32], [33], [34], [35]. Removing the local fusion restriction, and allowing vesicles to fuse with any cisterna and the ER depending on their SNARE concentrations, we achieve only poor enzyme segregation with all enzyme maxima shifted towards younger cisternae, (Fig. 4).

We suggest therefore, that a realistic description of fusion probability in *S. cerevisiae* must include a factor that considers fusion preferences related to cisternal age although it might be less stringent than the nearest neighbor limitation of a Golgi stack. Golgi scattering occurs when novel cisternae emerge from multiple, short-lived transitional ER (tER) sites rather than from a single, stable tER [36]. If individual tER sites release multiple cisternae in short succession before ceasing their activity, the diffusion limits imposed by the ER-network could maintain sister cisternae that are close in age in proximity to each other, thus *ad hoc* generating a series of maturing Golgi cisternae that remain separate from those generated in parallel by other tER sites. Evidence from a recent study by Nakano et al in *S. cerevisiae* supports this prediction: When due to altered ER-morphology the motility of Golgi elements away from the ER-exit site(s) is impeded, *cis* and *trans* Golgi elements could be seen in close proximity to each other and to ER-exit sites [37]. A position-age correlation is also apparent from the more coarse-grain viewpoint: Consider the emission of Golgi elements from multiple scattered ER exit sites and their subsequent one-dimensional diffusion in the cytoplasmic half-space away from ER membrane. The average distance from the ER membrane of a Golgi element at time t after emission scales as 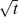. Thus, the older Golgi elements are on average further away from the ER than the younger ones. Real-time imaging maps of the spatial relationship between yeast Golgi cisternae that exchange cargo should provide the experimental framework to make our model applicable to Golgi systems with scattered compartments where we expect the enzyme distribution to be somewhere in between the examples shown in Fig. 3 and Fig. 4.

## Discussion

We present a simple model that explains the establishment and maintenance of directed vesicular flow and concentration gradients in the Golgi apparatus, an organelle system that undergoes constant rejuvenation by adding a new cisterna at the cargo-entering *cis* side while dissolving the oldest cisterna as secretory and lysosomal cargo exit at the *trans* end. Age is indeed the distinguishing feature of individual Golgi cisternae that we identify as the key to symmetry breaking. As cisternae mature the concentration of their functional SNAREs decreases, thereby providing the seed for a *cis* 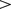 *trans* cisternal gradient of fusion factors for transport vesicles. This SNARE gradient causes the predominantly retrograde direction of vesicular flux that retrieves Golgi resident proteins, such as the SNAREs themselves and enzymes, from older to younger cisternae and back to the ER. The vesicular transport of SNAREs further enhances their gradient until a steady state between the retrograde vesicular and anterograde cisternal progression is reached. Both the seed gradient and cisternal maturation are indispensable for this outcome.

The “seeding” temporal decay of cisternal SNARE concentrations occurs via several mechanisms: i) Retrieval to the ER alone can account for the loss of the SNAREs present mostly in the young cisternae. However, the retrieval to the ER of the Golgi SNAREs from the medial and *trans* cisternae is not sufficient to create a seed gradient. ii) Experimental evidence for one such late Golgi SNARE, Gos28, are compatible with the notion that its loss occurs through degradation: The levels of Gos28 can go up as much as 40% when the availability of its chaperone GATE-16 is increased, preventing Gos28's proteolytic degradation [38], [39]. Gos28-levels also increase when components of the Golgi-tethering complex COG are overexpressed [40]. This adjustability means that a fraction of Gos28 is indeed wasted under the normal operational conditions. iii) Loss of SNAREs may also involve mechanisms in which Golgi-SNAREs become diverted to extra-Golgi functions. In yeast, Golgi-derived vesicles were shown to serve as source for autophagic membranes, which are later retrieved back to the Golgi [41], [42]. The Gos28 homologue Gos1p in particular, has been implicated in the retrieval of the autophagic membrane protein Atg9 to the Golgi [41]. iv) The loss of function of 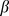 t-SNARE in older cisternae may occur due to modification of the membrane properties. v) A fraction of the decay of the late Golgi t-SNARE is due to its inactivation by the corresponding v-SNARE with its emerging counter-current gradient (see Fig. 3). Cognate SNARE complexes not only assemble when present on opposite membranes (i.e. in *trans*) but also when present at the same membrane (i.e. in *cis*), where most of them are disrupted under energy expenditure by the NSF/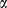
SNAP machinery [43], [24]. Nevertheless, in freshly isolated plasma membranes, where the v-SNARE concentration is low, about 10% of t-SNAREs are found in unproductive SNARE complexes [44]. As the v-SNARE concentration goes up from *cis*- to *trans*-Golgi (blue line in Fig. 3A) concomitant with the decreasing t-SNARE levels (green line in Fig. 3A), binding of the t-SNARE into fusion-incompetent SNARE complexes will sharpen the *cis* 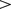 *trans* gradient of its fusion-competent concentration.

Once the retrograde vesicular flux is established, different affinities of Golgi enzymes for the vesicles explain the enzyme peaks in *cis*, medial and *trans* cisternae. One finding from our simulations is that the differential distribution of Golgi proteins can only be achieved when the vesicles are allowed to recycle back to the ER. This is in good agreement with experimental observations [45], [23], [29]. However, the importance of Golgi protein cycling through the ER for the enzyme segregation had not been appreciated in previous models that explained the Golgi enzyme peaks [10], [21] because of the arbitrarily implementation of the directionality of vesicle transport. It should be possible to test this important conclusion from our model experimentally. In yeast, ER- recycling of Golgi-derived vesicles can be stopped and the consequences for the segregation of *cis* and *trans* Golgi enzymes can be monitored by dual color time-lapse microscopy [33], [34]. This approach is feasible in strains harbouring temperature-sensitive mutations in ER-t-SNAREs [46], [47]. Importantly, the switch to the non-permissive temperature does not lead to the accumulation of Golgi-derived transport vesicles in these strains, presumably because ER-destined vesicles also contain significant amounts of 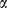 v-SNAREs, which allows them to efficiently fuse with the Golgi when fusion with the ER is thwarted. Such a scenario is indeed consistent with the SNARE dissociation constants of our model (Table 1).

Our simulations are insensitive to a broad spectrum of initial conditions. Regardless of whether we started with a single cisterna and added new cisternae one by one as it would occur during Golgi *de novo* formation, or considered a complete stack of identical cisternae when turning on the vesicular flux and SNARE loss mechanism, in each case the same steady state was reached.

An important question is the relevance and specificity of constants used for the modeling. Naturally, the range of admissible constants narrows as one reproduces more detailed and specific scenarios. Our first observation, that a temporal loss of SNAREs results in directed vesicular flux, is very general and holds for virtually any set of constants (see Eqs. (11, 12)). The selection of constants became more restrictive when the *cis*, medial, and *trans* peaks of Golgi enzymes and the actual 2 SNARE pair scenario were reproduced. The dissociation constants for binding of SNAREs and enzymes to vesicular sites had to be tuned within a 10% precision. The actual values of the dissociation constants are of the same order as protein concentrations, which is quite common for protein-protein interactions and appears to be evolutionally attainable [48]. Furthermore, to reproduce the shape of experimentally measured enzyme and SNARE peaks, the directionality of vesicular flux needed to be sufficiently strong, which we attempted to achieve while minimizing the decay term for SNAREs. The SNARE decay and vesicular transport constants did not have to be tuned as precisely as the dissociation constants and their admissible variation range is generally 20–30%.

We observed that the distinct enzyme peaks can be achieved with just one cognate SNARE pair. Why then does the Golgi afford two SNARE pairs? One proposal, put forward by Volchuk et al., is that only the 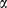 SNARE mediates retrograde transport of Golgi resident proteins while the 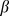 SNARE is dedicated to anterograde transport of exocytic and lysosomal cargo [17]. We consider this unlikely, however, based on the collective experimental evidence. Immuno electron microscopy-based observations of anterograde cargo in COPI vesicles is controversial and more recent organelle proteomics readily identified Golgi resident proteins but no exocytic cargo in COPI vesicles (reviewed in [49]). Moreover, functional data in yeast have provided unequivocal evidence for a role of the 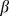 SNARE in Golgi enzyme trafficking. Thus, acute inhibition of the 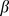 SNARE Sft1 leads to a rapid loss of *trans* and medial Golgi enzymes from Golgi cisternae and their dispersion into vesicles that are apparently unable to fuse [23]. Therefore, both SNARE pairs are likely to operate in tandem rather than in a countercurrent fashion. Although the concentration of vesicular SNAREs does not influence the directionality of fusion, it determines fusion efficiency. Thus, high concentrations of one of each v-SNAREs on either end of the Golgi can sustain efficient vesicular traffic throughout the Golgi stack. In addition, each SNARE pair could have distinct, additional roles at the Golgi boundaries. While this is well established only for the 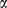 SNARE, which mediates fusion of ER-derived vesicles at the *cis* face (reviewed in [15]), recent evidence suggests that 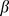 SNAREs GS15 and Ykt6 can participate in the fusion of endosomes with the *trans* Golgi or TGN [50].

According to our model, the experimentally observed steep *cis* 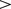 *trans* gradient of the 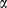 SNARE results in an almost sequential action of the two SNAREs within the maturing Golgi stack. This in turn yields two *de facto* distinct COPI vesicle populations, one enriched in 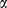 SNAREs and *cis* Golgi markers, the other in 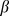 SNAREs and enzymes from the medial and *trans* Golgi. Plant Golgi stacks indeed feature morphologically distinct vesicles around the rim of the *trans* and *cis* cisternae, respectively [51] and in mammalian cells COPI vesicles enriched in either *cis* and *trans* Golgi proteins and the corresponding SNAREs have been distinguished [52], [8], [53]. In our model these two subpopulations of COPI vesicles are simply due to differences in the competitiveness of the SNAREs and enzymes for binding to a universal COPI-coat rather than to two vesicle types that differ in the composition of the COPI coat or, more generally, in the machinery for cargo selection. Even though vertebrates have been reported to possess several COPI isoforms [54], we show that a single COPI species, as in fungi and plants, is sufficient generate the variance in vesicle content.

In summary, we have presented an explanation for why the minimal requirement of one SNARE pair and one vesicle type for the generation and maintenance of each distinct organelle [14] is relaxed for organelles that evolve from each other through maturation. Apart from the Golgi apparatus this might also be relevant for the organelles along the plasma membrane-early endosome-late endosome axis.

## Methods

### Establishment of a *cis* 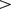 *trans* SNARE gradient that mediates retrograde vesicular flow

Here we do not specify the nature of t- and v- SNAREs, simply calling fusiogenic molecules present in a vesicle and cisterna v-SNAREs and t-SNAREs. The chemical distinction between t- and v-SNAREs will be stated later. However, to keep the same notations throughout the paper, we use the specific 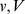 and 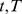 notations already here. Small letters denote the vesicular concentrations of a molecule with the subscript referring to the parental cisterna. So 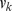 and 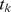 are concentrations of v- and t-SNAREs, and 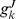 is the concentration of the 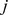
th Golgi enzyme in a vesicle that emerged from the 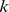
th cisterna. Capital letters 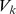, 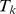, and 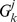 denote the concentrations of these substances in cisterna number 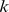. We number the compartments in the *cis* to *trans* direction, so the youngest cisterna has number one.

The number of vesicles that bud from the 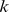
th compartment per unit time, 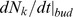, is assumed to be proportional to the area of the compartment 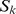,
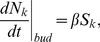
(1)where 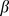 is the budding rate constant which depends on the concentration and activity of coat proteins.

A vesicle emitted from the 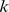
th cisterna fuses with the 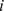
th cisterna with a probability proportional to the product of the concentrations of the SNARE in the vesicle 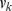 and the SNARE in the cisterna 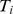. The number of vesicles that fuse with the cisterna 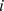 per unit time is
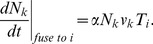
(2)with 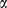 being the fusion rate constant. The assumption of local transport restricts a vesicle emitted by the 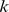
th cisterna to fuse with the 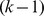
th, 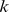
th, and 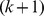
th cisternae. The *trans*-most cisterna does not receive any retrograde vesicular input. The time evolution of the population of vesicles emitted by the 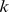
th compartment is described by the rate equation which includes both the budding and fusion terms.
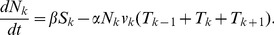
(3)At steady state, the concentration of vesicles emitted by the 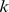
th compartment becomes
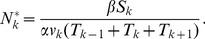
(4)Hence, an increment in SNARE concentration in the 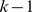
st cisterna due to the vesicular flux from the 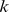
th cisternae is
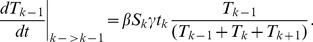
(5)A dimensionless factor 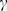 describes how the cargo is “diluted” when a vesicle fuses with its target cisterna and is equal to the ratio of vesicle to compartment surface areas. Assuming mass-action equilibrium between the vesicular binding sites and its cargo (t-SNARE) and that budding of a single vesicle does not significantly alter the cisternal concentration of t-SNARE, the amount of t-SNARE in a vesicle is
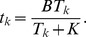
Here 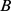 is the concentration of cargo binding sites in a vesicle and 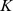 is the dissociation constant for binding between cargo and such sites. Eq. (5) indicates that the steady state flux of vesicles does not depend on the v-SNARE concentration and is only determined by the budding rate and t-SNARE distribution. In the following we assume that the volume and the budding area of the compartments remains constant, 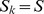. Relaxing this assumption does not substantially change the results.

The rate equation that describes the evolution of the t- SNARE concentration in the 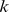
th compartment reads
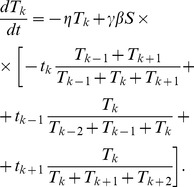
(6)The first term describes the loss of the t-SNARE with the per molecule rate 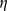. To keep it general, the loss term collects all mechanisms of t-SNARE decay approximately described by first-order kinetics, such as degradation, loss of mis-targeted vesicles, etc. Thus, here the lost vesicles are not accounted for in Eqs. (3, 4), but are only included in the first term in Eq. (6). The second line describes the outgoing vesicular transport from the 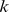
th cisterna to its 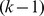
th and 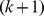
th neighbors, and the last two lines represents the incoming flux from the same neighbors to the 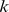
th cisterna.

To complete the description of t-SNARE distribution, the vesicular transport equation (6) has to be complemented by the definition of cisternal dynamics: Every 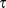 time units the running number of each cisterna is incremented by one, so that the 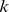
th cisterna becomes 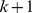
st. A new first cisterna with a given initial concentration of t-SNARE 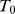 is added to the *cis* end of the stack, while the *trans*-most cisterna is removed.

We measure cisternal concentrations of SNAREs and other molecules in the units of their initial concentrations in the first cisterna and the natural unit of time is the period of cisternal maturation 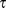. This is equivalent to setting these quantities equal to one. Then the t-SNARE distribution is described by three parameters: decay rate 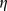, the vesicular transport coefficient 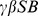, and the dissociation constant 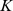.

This cisternal maturation scenario together with Eq. (6) are implemented numerically as a simple Euler update. For reasonable values of parameters the system quickly converges to a steady regime: In each cisterna concentrations of t- and v-SNAREs undergo periodic evolution with the period 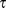. Plots of the cisternal distributions of the t-SNARE are presented in Fig. 2A in the Results.

### Analytic solution for the asymptotic steady state cisternal concentrations of SNAREs

Consider a hypothetical system where the number of cisternae is non-biologically large. For older cisternae, the concentrations of SNAREs are small, 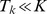, so the uptake of a SNARE into a vesicle is proportional to the concentration of the SNARE in the progenitor cisterna, 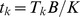. In the asymptotic regime, i.e., sufficiently far from the first and the last cisterna, we seek a solution of Eq. (6) in the form
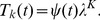
(7)After substitution into (6) it yields
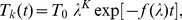
(8)where
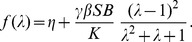
(9)We look for the periodic solution in a sense that each 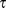 time units, after the addition of a new cisterna and dissolution of the most mature cisterna, the system returns to the same state. So the 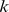
th cisterna at the time 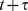 must be identical to the 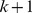 cisterna at the time 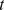,
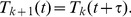
(10)This yields the following equation for 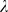,
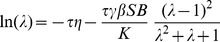
(11)which is solved numerically.

**Figure 5:**
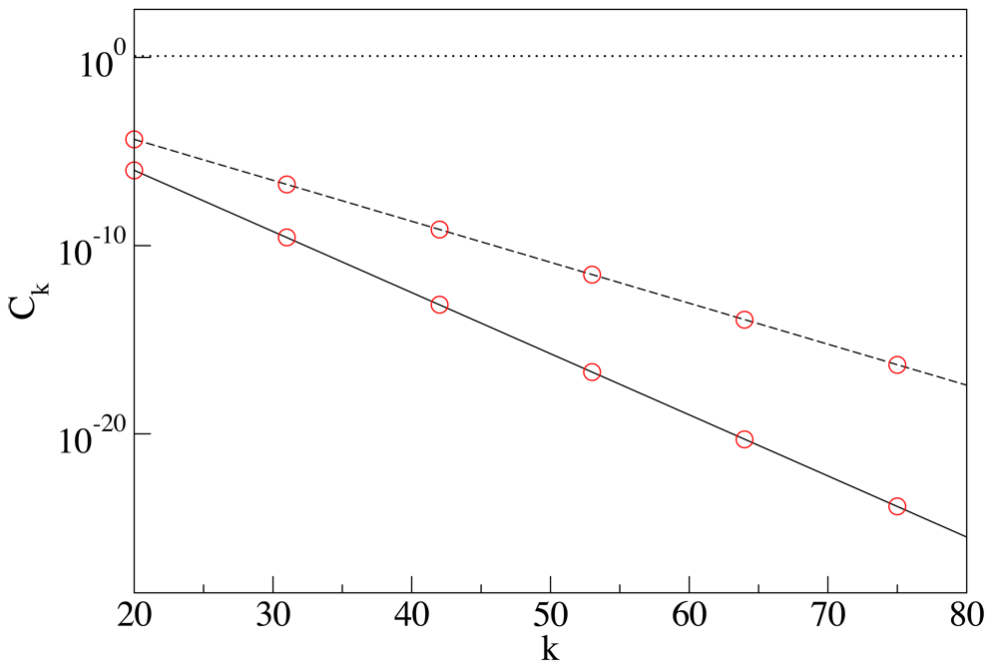
Distribution of SNARE for large number of cisternae. The steady state gradient has the exponentially decaying form, 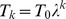 where 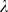 depends on two dimensionless groups of parameters, 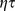 which caracterizes the decay of the SNARE and 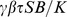 which characterizes the vesicular transport of SNARE: 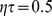 and 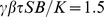 (solid line) with the best fit given by 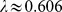, 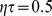 and 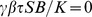 (dashed line) with the best fit given by 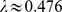. Theoretical values of 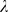 determined from (11) are indistinguishable from the values obtained as the best fit for the simulations. The dotted line corresponds to the case of zero loss, 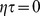 and 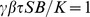, and illustrates the absence of a concentration gradient. To reveal the exponential decay of the SNARE concentration, we purposefully consider a non-biologically high number of compartments and analyze the SNARE concentration away from both the *cis* and *trans* ends of the stack, where the boundary effects can play a role.

We observe that in the asymptotic regime, the steady state t-SNARE concentration decays exponentially with the number of cisterna,
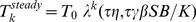
(12)with the coefficient 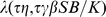 being the solution of Eq. (11). Simulations confirm our theoretical prediction given by (7, 11), see Fig. 5.

The necessity of the loss term for establishing the gradient by breaking the initial symmetry between the cisternae is clearly revealed by the following analytic argument: For a small loss rate (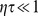), the expansion of the steady state exponent 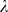 reads
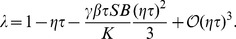
(13)Hence 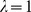 for 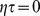 independent of the intensity of the vesicular transport characterized by 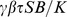. Indeed, without breaking the initial similarity between cisternae, a vesicle would fuse with any of its three target compartments with the same probability, so that vesicular flux into a compartment would be equal to the vesicular flux out of this compartment. In other words, the vesicular transport can only enhance the initial difference in concentrations between cisternae, created by some other mechanism, rather than create this difference *de novo*.

### Establishment of Golgi enzyme peaks in *cis*, medial and *trans* cisternae via SNARE-mediated retrograde vesicular traffic

The transport of Golgi enzymes with cisternal concentrations 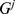, where 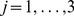 labels an enzyme class, is described by an equation analogous to Eq. (6). The difference is that instead of a single vesicular cargo type (t-SNARE), we now consider three classes of competitors for vesicular seats. Thus, for each 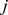, 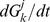 replaces the 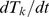 in the left-hand side and 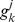 replaces 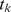 in the right-hand side of Eq. (6),
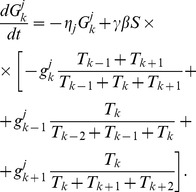
(14)Here (only in this subsection) we assume that the enzyme transport does not affect the vesicular flow, which is established by the autonomously evolving t-SNARE distribution, described by (6). The concentration of enzyme 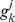 uploaded to a vesicle is determined by the mass action equilibrium
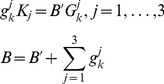
(15)Here 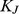 are vesicle-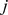
th enzyme dissociation constants, the last equation states that the total number of the vesicular binding sites 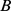 is equal to the number of free sites 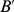 plus the number of sites occupied by enzymes of all three classes. Solving (15), one finds 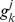,
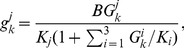
(16)which are subsequently substituted into Eq. (14), As with SNAREs, each newly formed (first) cisterna is assumed to be loaded with Golgi enzymes with given concentrations, 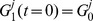 We assume that there is no temporal decay of enzymes, so 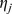 is put equal to zero in the transport equations.

**Figure 6:**
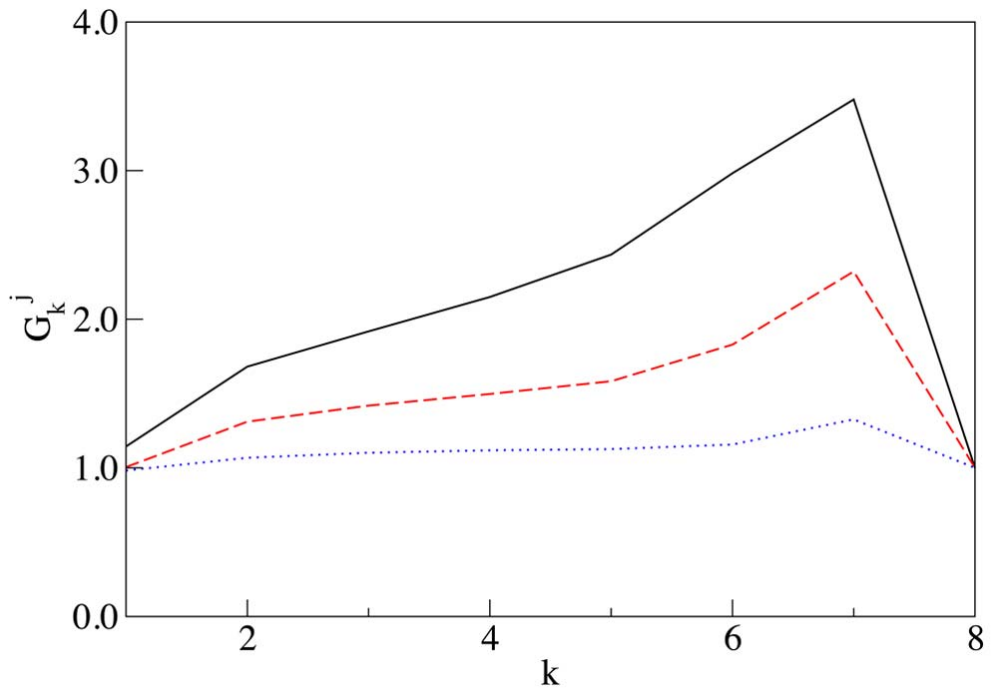
Enzyme segregation depends on the open boundary condition. Steady state distribution of the same enzymes as in Fig. 2 if the “Closed boundary conditions” on the *cis* end of the stack are assumed: No vesicles can exit the Golgi. The parameters are the same as in Fig. 2.

When the retrograde vesicular transport is counterbalanced by the anterograde cisternal progression, the enzyme distribution reaches its steady state. The nature of the steady state depends on the boundary conditions imposed on the *cis* side of the Golgi stack: An “open” boundary condition is implemented as a zeroth cisterna (ER) with a fixed concentration of t-SNAREs which can fuse vesicles (see Fig. 2B), while under the “closed” boundary conditions vesicles do not escape the Golgi, (see Fig. 6).

### The two Golgi SNARE pairs can function with a single vesicle type to establish their own gradients and the observed Golgi enzyme peaks in *cis*, medial and *trans*

Putting together the two mechanisms considered above, we introduce a realistic model of Golgi transport. It describes the evolution of 8 distinct types of molecules: 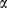 and 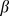 sets of t- and v-SNAREs controlling intra-Golgi fusion, a v-SNARE for fusion with the ER, and *cis*, medial, and *trans* types of enzymes. For brevity of equations, we use the universal notations 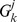 and 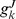 for cisternal and vesicular concentrations of each of the eight molecules, 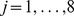. At the same time, in the fusion rate terms we retain the specific notations for t- and v-SNAREs with superscripts “
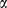
”, “
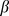
” and “ER” denoting the affiliation of particular SNAREs. The evolution of the cisternal concentration 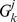, 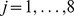 of each type of molecule is described by the rate equation similar to (14) with two important distinctions. First, the rate of fusion of a vesicle with a cisterna, previously given by (2), is now proportional to the sum of the products of the concentrations of *cis* and *trans* SNAREs [14].
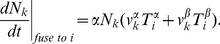
(17)The increment in the vesicular cargo concentration in the 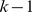
th cisterna due to the vesicular flux from the 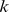
th cisternae is (compare to (5)),
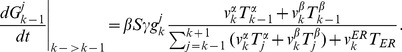
(18)Here 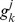 is the vesicular concentration of molecule 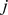 defined by mass-action equilibrium (16) between vesicular binding sites and all eight competing molecules. The last term in the denominator corresponds to the fusion of vesicles with the ER, which is the second distinction of the considered mechanism from the model case analyzed above. The ER t-SNARE concentrations 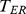 is considered to remain constant and vesicles originating from any cisterna can fuse with the ER. Assembling together all gain and loss mechanisms for the cisternal concentration of 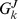, we write the complete system of kinetic equations that describe the vesicular transport.
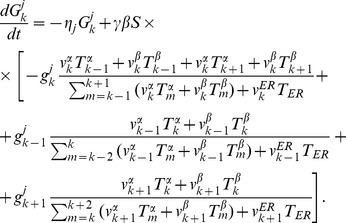
(19)


The escape of a fraction of vesicles from the Golgi to the ER provides one part of a loss mechanism necessary for seeding the gradient of t-SNAREs. Yet we do not exclude the possibility of other mechanisms of t-SNARE decay, so the 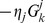 remains present in the rate equation. In the simulations, we set 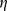 for the 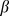 t-SNARE equal to a small value, while the decay coefficients for all other substances are put equal to zero.

### Vesicular transport without nearest neighbor fusion restriction

To model the vesicular transport in yeast, we used an equation similar to Eq. (19) where the restriction of nearest neighbor fusion was relaxed,
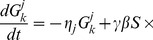
(20)

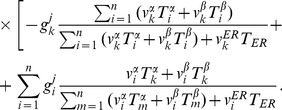
(21)A typical steady state distribution of enzymes produced by the unrestricted vesicular fusion is shown in Fig. 4.
